# Syllabus of the Obstetrical Lectures in the Medical Department of the University of Pennsylvania

**Published:** 1891-12

**Authors:** 


SYLLABUS OF THE OBSTETRICAL LECTURES IN THE MEDICAL DEPARTMENT OF THE UNIVERSITY OF PENNSYLVANIA. By RICHARD C. NORRIS, A. M., M. D., Demonstrator of Obstetrics, University of Pennsylvania; Physician to the Methodist Episcopal Hospital; Obstetrical Registrar Philadelphia Hospital. Second edition. Philadelphia: W. B. Saunders. 1891. Price, $2.00.

This work, the author states, is a necessity. The difficulty in note-taking in medical lectures is recognized by all medical students. In obstetrics, through the use of the manikin and plates to illustrate the subject, the difficulty complained of cannot be as great as in other branches, but even here the author attempts to aid the student, by furnishing the salient points of each lecture, arranged in a consecutive order, with a view to assist in the subsequent study of the subject. After a careful examination, we think the work will subserve a good purpose. It contains a vast amount of well-digested matter, thoroughly condensed and clearly presented. Teachers realize that in the pressure and work of the session, in which the student has but a limited time to devote to the many subjects to which he listens daily; thus he will find in this little book an invaluable aid for his preparation for quizzes and examinations.

The author has performed the task assigned him, in giving a digest of Prof. Hirst's lectures, in a manner creditable alike to himself and to the teacher whose lectures he aims to epitomize.

